# Sex-Specific Differences in Pathogen Susceptibility in Honey Bees (*Apis mellifera*)

**DOI:** 10.1371/journal.pone.0085261

**Published:** 2014-01-17

**Authors:** Gina Retschnig, Geoffrey R. Williams, Marion M. Mehmann, Orlando Yañez, Joachim R. de Miranda, Peter Neumann

**Affiliations:** 1 Swiss Bee Research Centre, Research Station Agroscope Liebefeld-Posieux ALP-HARAS, Bern, Switzerland; 2 Institute of Bee Health, Vetsuisse Faculty, University of Bern, Bern, Switzerland; 3 Department of Biology, Dalhousie University, Halifax, Nova Scotia, Canada; 4 Department of Ecology, Swedish University of Agricultural Sciences, Uppsala, Sweden; 5 Social Insect Research Group, Department of Zoology and Entomology, University of Pretoria, Pretoria, South Africa; University of Arizona, United States of America

## Abstract

Sex-related differences in susceptibility to pathogens are a common phenomenon in animals. In the eusocial Hymenoptera the two female castes, workers and queens, are diploid and males are haploid. The haploid susceptibility hypothesis predicts that haploid males are more susceptible to pathogen infections compared to females. Here we test this hypothesis using adult male (drone) and female (worker) honey bees (*Apis mellifera*), inoculated with the gut endoparasite *Nosema ceranae* and/or black queen cell virus (BQCV). These pathogens were chosen due to previously reported synergistic interactions between *Nosema apis* and BQCV. Our data do not support synergistic interactions between *N. ceranae* and BQCV and also suggest that BQCV has limited effect on both drone and worker health, regardless of the infection level. However, the data clearly show that, despite lower levels of *N. ceranae* spores in drones than in workers, *Nosema*-infected drones had both a higher mortality and a lower body mass than non-infected drones, across all treatment groups, while the mortality and body mass of worker bees were largely unaffected by *N. ceranae* infection, suggesting that drones are more susceptible to this pathogen than workers. In conclusion, the data reveal considerable sex-specific differences in pathogen susceptibility in honey bees and highlight the importance of ultimate measures for determining susceptibility, such as mortality and body quality, rather than mere infection levels.

## Introduction

Sex-specific phenotypic differences can influence susceptibility to various stressors encountered in the environment such as pathogens or toxins [1]. Susceptibility, defined as the likelihood to develop ill effects from an external agent [2], can be measured using multiple indices. For example, susceptibility to a pathogen can not only refer to host mortality, but also to sub-lethal responses (*e.g.* reduced body mass), severity of infection (infection level susceptibility), or likelihood of infection (infection or disease susceptibility).

Differential susceptibility to pathogens between sexes is well known in numerous species, particularly in vertebrates [3]–[4]. For invertebrates, Morton and García del Pino (2013) demonstrated that in house crickets (*Acheta domesticus*), American cockroaches (*Periplaneta americana*), and flatheaded rootborers (*Capnodis tenebrionis*), males exhibited higher immunocompetence to pathogens than females [5]; the opposite was observed for crickets (*Gryllus texensis*) and scorpionflies (*Panorpa vulgaris*) [6]–[7]. Differences in immunocompetence between sexes are likely the result of complex investment strategies that differentially partition limited resources to vital functions and processes, including reproduction [8]. For species exhibiting haplo-diploidy, the haploid susceptibility hypothesis proposes that the absence of heterozygosity at immune loci in haploid individuals may be responsible for differential immunocompetence between haploid and diploid individuals [9]. Ruiz-González and Brown (2006) reviewed seven studies that examined the impact of the pathogens *Crithidia bombi*, *Nosema bombi*, or *Locustacarus buchneri* in various bumble bee (*Bombus* spp.) species [10]. Haploid males had lower parasite prevalence than diploid females in 15 of 26 investigated host-parasite combinations.

This study investigated sex-specific differences in pathogen susceptibility in the western honey bee (*Apis mellifera*). The honey bee is the most important pollinator of agricultural crops [11]–[12], and has over the last several decades suffered increasingly severe colony deaths in many regions of the northern hemisphere [13]. Although the reasons for increased colony mortality are not fully understood, it is likely that multiple stressors, acting either alone or in combination, are to blame. These include changing agricultural practices, beekeeper management issues, as well as introduced parasites and pathogens [14]–[17].

Honey bee microfloral communities consist of numerous viruses, fungi, microsporidia and bacteria [18]–[23]. This simultaneous co-infection offers a plethora of opportunities for inter-specific microbial interactions that could be symbiotic (co-dependence), synergistic (mutually or unilaterally facilitating), or antagonistic (mutually or unilaterally inhibitory), which could have considerable influence on both pathogen distribution and virulence [24]. There are a number of examples of such interactions among honey bee parasites and pathogens. For example, the midgut microsporidian *Nosema apis* is a facultative requirement for successful infection of black queen cell virus (BQCV) and an obligatory requirement for bee virus Y (BVY) infection [25]. Similarly, bee virus X (BVX) is associated with, and partially dependent on, co-infection with the protozoan *Malphigamoeba mellifica* [25]. Conversely, a negative association was observed between *Nosema ceranae* and deformed wing virus (DWV) in the honey bee midgut, although not for whole bees or at colony level [26]–[27]. Further examples of antagonistic interactions include those between lactic acid bacteria and both *Paenibacillus larvae* [28]–[30] and *Melissococcus plutonius* [31]; causative agents of American foulbrood (AFB) and European foulbrood (EFB), respectively.

Most studies investigating interactions among honey bee pathogens have focussed on workers and queens [26], [32]–[34] rather than on drones [35]. Drones are a critical element of both apicultural breeding and natural colony fitness selection [36]. They exhibit substantial differences in behaviour and physiology compared to workers or queens [37] that may influence their susceptibility to pathogens. Although drones have been the subject of numerous studies relating to their reproductive and genetic functions [38]–[42], relatively little is known about the impact and interactions between common honey bee pathogens in drones, and even less about differential disease susceptibility between the honey bee sexes. Drones are much more susceptible than workers to *Varroa destructor*, a parasitic mite that reproduces in brood [43]–[44], but less susceptible to deformed wing virus, which is vectored by the mite, at least when comparing live (surviving) drones and workers [45]. Both drones and workers are equally susceptible to *N. apis* infection [46]. However, corresponding data for *N. ceranae*, a similar pathogen, is currently lacking. Both are widespread gut pathogens [46]–[47] transmitted horizontally via the faecal-oral route [48], inflicting gut tissue damage [49] and suppressing the honey bee immune response [50]–[51], thereby possibly promoting viral infections and reducing honey bee longevity [46]. BQCV is one such *Nosema*-associated virus [25]. This virus also infects mid-gut tissues and can be transmitted via the faecal-oral route but does not cause visible symptoms in infected adult honey bees [52] and to date, very little is known about potential effects on honey bee health. BQCV is closely associated with *N. apis* infection [25] but how it interacts with *N. ceranae* is still unclear. Nothing is known about sex-specific differences in susceptibility to BQCV between drone and worker bees.

The aim of these experiments was to assess the susceptibility of workers and drones to *N. ceranae* and BQCV, as well as their inter-specific interaction, using common indices of honey bee health. We used host mortality (e.g. [33]), body mass (e.g. [17]) and pathogen infection level as measures of susceptibility. The experiments were conducted using standardized laboratory hoarding cages, in order to maximize control over the infection process and the environmental conditions.

## Experimental Procedures

### Ethics statement

No animal use protocol was required by the Veterinary Office of the district of Bern or the Federal Veterinary Office to perform this research on honey bees. No endangered or protected species were involved in the study. Privately owned land was used and accessed only after permission from the landowner.

### Experimental design

The experiment was conducted during summer 2011 at Agroscope Liebefeld-Posieux Research Station ALP-HARAS in Bern, Switzerland, and consisted of 80 cm^3^ disposable hoarding cages, each containing either 10 drones plus 10 attendant workers or 20 workers. The cages were assigned to a fully-crossed design of *N. ceranae* and BQCV treatments (Table 1), with four and five replicate cages per treatment group for the drone-plus-workers and the workers-only experimental groups, respectively. The 1∶1 drone:worker ratio for the drone part of the experiment was adapted from prior recommendations of 0.5∶1 [53], 1.5∶1 [46] and 2∶1 [54] drones∶workers, to ensure adequate attendance of the drones.

**Table 1:** Treatment groups and schedule of the cage experiments.

Treatment group	Inoculated at day 0	Sample size & reps.	Inoculated at day 7	Sample size & reps.	Day 14	Sample size & reps.
**Drones**						
Control	Control suspension	N = 10, 4 reps.)	Control suspension	(N = 7–8×4 reps., total 30)	Termination at −20°C	(N = 2–5×4 reps., total 15)
BQCV	Control suspension	(N = 10, 4 reps.)	**BQCV** suspension	(N = 7–10×4 reps., total 36)	Termination at −20°C	(N = 0–7×4 reps., total 13)
*N. ceranae* & BQCV	***N. ceranae*** suspension	(N = 10, 4 reps.)	**BQCV** suspension	(N = 6–9×4 reps., total 30)	Termination at −20°C	(N = 0–1×4 reps., total 3)
*N. ceranae*	***N. ceranae*** suspension	(N = 10, 4 reps.)	Control suspension	(N = 4–9×4 reps., total 29)	Termination at −20°C	(N = 0–3×4 reps., total 5)
**Workers**						
Control	Control suspension	(N = 20, 5 reps.)	Control suspension	(N = 18–20×5 reps., total 96)	Termination at −20°C	(N = 15–19×5 reps., total 86)
BQCV	Control suspension	(N = 20, 5 reps.)	**BQCV** suspension	(N = 16–20×5 reps., total 95)	Termination at −20°C	(N = 14–20×5 reps., total 87)
*N. ceranae & BQCV*	***N. ceranae*** suspension	(N = 20, 5 reps.)	**BQCV** suspension	(N = 18–20×5 reps., total 94)	Termination at −20°C	(N = 16–19×5 reps., total 88)
*N. ceranae*	***N. ceranae*** suspension	(N = 20, 5 reps.)	Control suspension	(N = 17–20×5 reps., total 94)	Termination at −20°C	(N = 16–18×5 reps., total 84)

The *N. ceranae* inoculum contained 100,000 *N. ceranae* spores in 50% (w/v) sucrose solution. The control bees received the *N. ceranae*-free suspension as well in 50% (w/v) sucrose solution. The pupae extract from BQCV-free pupae was administered in 50% (w/v) sucrose solution and BQCV inoculum was composed of highly concentrated BQCV suspension of infected pupae in 50% (w/v) sucrose solution. Each bee was individually inoculated with 5 µl of the respective treatment suspensions (reps.  =  replicates).

### Source of honey bees

Experimental honey bees (*A. mellifera*) were obtained by collecting brood frames from 3 queen-right colonies during the local mating season (June 2011). Those colonies had low *V. destructor* infestation levels (<3 mites per 100 bees), as determined using the soapy water wash method [55].

To obtain uniformly aged drones, the queens of the 3 colonies were confined for 2 days to drone frames. Close to emergence the frames were removed and housed individually in a wood and glass frame cage in an incubator with typical brood nest conditions [56] of 34.5°C, ≥50% relative humidity and near total darkness [57]–[58]. The frames were removed at 2 hour intervals to detect drone emergence. When antennae appeared, the wax caps were carefully removed manually using blunt forceps and the emerged drone and its respective brood cell were inspected with an LED light for the presence of *V. destructor*. Drones that were exposed to *V. destructor* during development were discarded. Drones that developed in cells without *V. destructor* were collected in a large 600 cm^3^ metal hoarding cage together with equal numbers of newly emerged workers from the same colonies. When enough drones were collected for the entire experiment, they were randomly assigned to the experimental cages and treatment groups. A separate cage containing workers of the same age and origin as the drones was kept in the incubator under identical conditions throughout the experiment to replace any dead attendant workers in the drone experimental cages.

To obtain experimental worker bees, two brood frames from the same colonies supplying the experimental drones were placed in a metal and glass frame cage in the incubator as described above. Every 4–10 h, newly emerged workers were carefully collected from the frames using forceps, inspected individually for *V. destructor* and transferred to a large 600 cm^3^ metal hoarding cage. Only non-infested workers were included in the cage experiment. Since *V. destructor* mites have >8-fold higher preference for drone brood than for worker brood [44], [59]–[60], the emerging drone brood was checked more intensively for mite infestation than the emerging worker brood. All experimental bees from the three source colonies were mixed and randomly allocated to their experimental cages within 2 days after collection. During this time they were maintained in large metal hoarding cages at incubator conditions and were supplied *ad libitum* with 50% sucrose solution.

### Source of pathogens

*Nosema ceranae* spores were obtained by preparing an extract in water from the midguts of six highly infected foragers [61] collected from the entrances of two *Nosema*-infected colonies located at the local research apiary. Spores were counted using a haemocytometer (Thoma, L.O. Labor Optik) and the extract was diluted to 20,000 spores per µl. The spores were identified as *N. ceranae* by qualitative PCR using a set of species-specific *Nosema* primers [62]. Briefly, genomic DNA of crushed midguts (in water) was extracted using the Nucleospin® Tissue kit (Macherey-Nagel) according to the manufacturer guidelines. The PCR was then performed using the Goldstar® DNA Polymerase (Eurogentec). The PCR involved 2 min at 94°C, 30 cycles of 30 s at 94°C, 30 s at 56°C, 30 s at 72°C and 7 minutes at 72°C. A control extract was prepared from the midguts of six non-infected adult bees that originated from the same two colonies and were found to be free from *N. ceranae*.

The BQCV inoculum was prepared by propagating a 10^−4^ dilution of a BQCV reference isolate [63] in 150 white-eyed honeybee pupae and preparing a chloroform-clarified extract in 10 mM phosphate buffer (pH 7.0)/0.02% diethyl dithiocarbamate, as described in de Miranda et al., (2013) [64]. The inoculum contained ∼1.4×10^9^ BQCV genome copies per µl extract and had no detectable contamination with ABPV, KBV, CBPV, DWV, VDV-1, LSV-1 and LSV-2; negligible (<0.0001%) contamination with IAPV and SBV, and <1% contamination with SBPV, as determined by RT-qPCR using the methods of Locke et al., (2012) [65]. A control extract was prepared from non-inoculated pupae. None of the viruses could be detected in this control extract, except BQCV (∼1.5×10^3^ copies/µl) and SBV (∼2.7×10^8^ copies/µl).

### Inoculation, incubation and sampling

For both the drone and worker part of the experiment, individual experimental drones and workers (but not the attendant workers in the drone cages) were each inoculated orally at 0 d with either 5 µl of the *N. ceranae* spore suspension (*i.e.* 100,000 spores per bee) or control suspension, both as 50% w/v sucrose solutions, using micropipettes (Table 1). Individuals were starved for 2 h before inoculation; those that did not consume the entire inoculum were discarded. Immediately after inoculation, workers were kept separated for approximately 20 min, the required time for the spores to be transported far enough in the intestinal system to avoid the transfer of the inoculum via trophallaxis [61], [66]–[67]. This precaution was not necessary for the drones because they are only recipients during trophallaxis [68].

BQCV inoculation took place 7 days after *Nosema* inoculation. Surviving individual experimental drones and workers (but not the attendant workers in the drone cages) each received an oral inoculum at 7 d of either 5 µl BQCV suspension (1 µl of pure extract in sucrose solution, *i.e.* 1.4×10^9^ BQCV genome copies/bee) or control extract, both as 50% w/v sucrose solution (Table 1). After inoculation with BQCV the experimental drones and workers were again isolated for approximately 20 min to prevent transfer of the inoculum through trophallaxis [61].

The bees in the experimental cages were fed 50% (w/v) sucrose solution *ad libitum*, using 1.5 ml Eppendorf tubes containing a single basal perforation, as well as gamma-irradiated sterilized pollen paste (10% pollen, 60% sugar, 30% water in 0.2 ml tubes with a 0.8 mm opening) for the duration of each assay. The cages were maintained in an incubator in complete darkness at 30°C and ≥60% relative humidity, reflecting natural hive conditions [56]. At 14 d, all surviving individuals were frozen and stored at −20°C until further analyses [69].

### Host mortality and body mass

Dead individuals were removed daily from their cages, recorded and stored at −20°C. For the drone experiment, a 1∶1 drone∶worker ratio was maintained throughout the experiment by removing or adding attendant workers as required.

The body mass was determined at the end of the experiment, on day 14, for the surviving experimental drones (total *n* = 15, 13, 3, 5 for control, BQCV, *N. ceranae* & BQCV, *N. ceranae* treatment groups, respectively) and a subset of the experimental workers (*n* = 25 per treatment group). For both drones and workers, only live bees were collected for weighing, thereby excluding the possibility to dry out after death. Each bee was weighed individually to the nearest 0.1 mg using an analytic scale (Mettler Toledo AT400).

### Nosema ceranae species confirmation and quantification

For both the drone and worker parts of the experiment, *N. ceranae* spore amounts were quantified in randomly selected individuals terminated at 14 d (*n* = 5 per cage). If fewer than five live drones were available on 14 d, those drones that died immediately prior to termination on 14 d were also included for the pathogen analyses (total *n* = 16, 13, 7, 9 for control, BQCV, *N. ceranae* & BQCV, *N. ceranae* treatment groups, respectively). Each drone or worker abdomen was homogenised in a 2 ml Eppendorf tube using a bead mill homogeniser (MM300 Retsch), one metal bead and either 300 or 250 µl TN buffer, for drones and workers respectively. Each homogenate was diluted to 1 ml prior to spore quantification, which was done according to Cantwell (1970) [70] using a haemocytometer (Thoma, L.O. Labor Optik) and a light microscope (Laborlux K, Leitz Wetzlar, Germany).

### RNA extraction and BQCV qPCR assays

For both the drone and worker parts of the experiment, BQCV was analysed in the same individuals as used for the *N. ceranae* quantification. Total RNA was extracted from aliquots of the homogenized abdomen suspension of each individual using the Nucleospin® RNA II kit (Macherey-Nagel) according to the manufacturer's guidelines. The extracted RNA was eluted in 50 µl of RNAse-free water. Reverse transcription was performed in 20 µl final volume using 10 µl of extracted RNA (2.5 ug), 200 ng of random hexamer primers using the Thermoscript™ RT system (Invitrogen), following the manufacturer guidelines. Before qPCR amplification, the cDNAs were diluted 10-fold in nuclease-free water. Each diluted cDNA sample was amplified in triplicate by qPCR using the KAPA SYBR® FAST Universal Mastermix kit (KAPA, Biosystems) in a RotorGene-3000A thermocycler (Corbett Research). Specific primers were used for BQCV (sequence 5′ - 3′: F: CGA CAG CGT GCC AAA GAG A, R: CGC CCA GCT TTG AAA CAG A) and for the honey bee β-Actin gene (sequence 5′ - 3′: F: CGT GCC GAT AGT ATT CTT G, R: CTT CGT CAC CAA CAT AGG), for virus and reference gene RNA quantification, respectively. The qPCR cycling conditions consisted of 3 min at 95°C, for enzyme activation, followed by 40 cycles of: 3 s at 95°C, for denaturation; 40 s at 60°C for annealing and extension, followed by fluorescence reading. Each run contained a 10-fold standard dilution series for both BQCV and the honey bee β-Actin gene. Runs were analysed using the programme LinRegPCR (HFRC, NL, v. 11.1). BQCV titres were normalised with those of the correspondent β-Actin. The data were converted to genome copies per bee by accounting for the various dilutions used in the cDNA preparation.

### Statistical analyses

Survival analyses were performed in SPSS (IBM SPSS Statistics 19) using Kaplan Meier Log-Rank for censored data, since some individuals were terminated at 14 d. Comparison of body mass as well as *N. ceranae* and BQCV amounts among treatment groups were performed in R (R Foundation for Statistical Computing, version 2.15.2012-09-19) using ANOVA to detect overall differences, as well as Tukey's HSD for multiple comparisons within treatment groups. *Nosema ceranae* and BQCV amounts were square root- and log10-transformed, respectively, to improve fit to normality prior to parametric statistical tests. For all statistical analyses, a significance level of α = 0.05 was applied. For analysis of the inter-specific interactions, parametric Pearson test for correlations in SPSS (IBM SPSS Statistics 19) using log10-transformed BQCV and non-transformed *N. ceranae* data (normally distributed in this treatment group) was used.

## Results

### Host mortality and body mass

**Figure 1:**
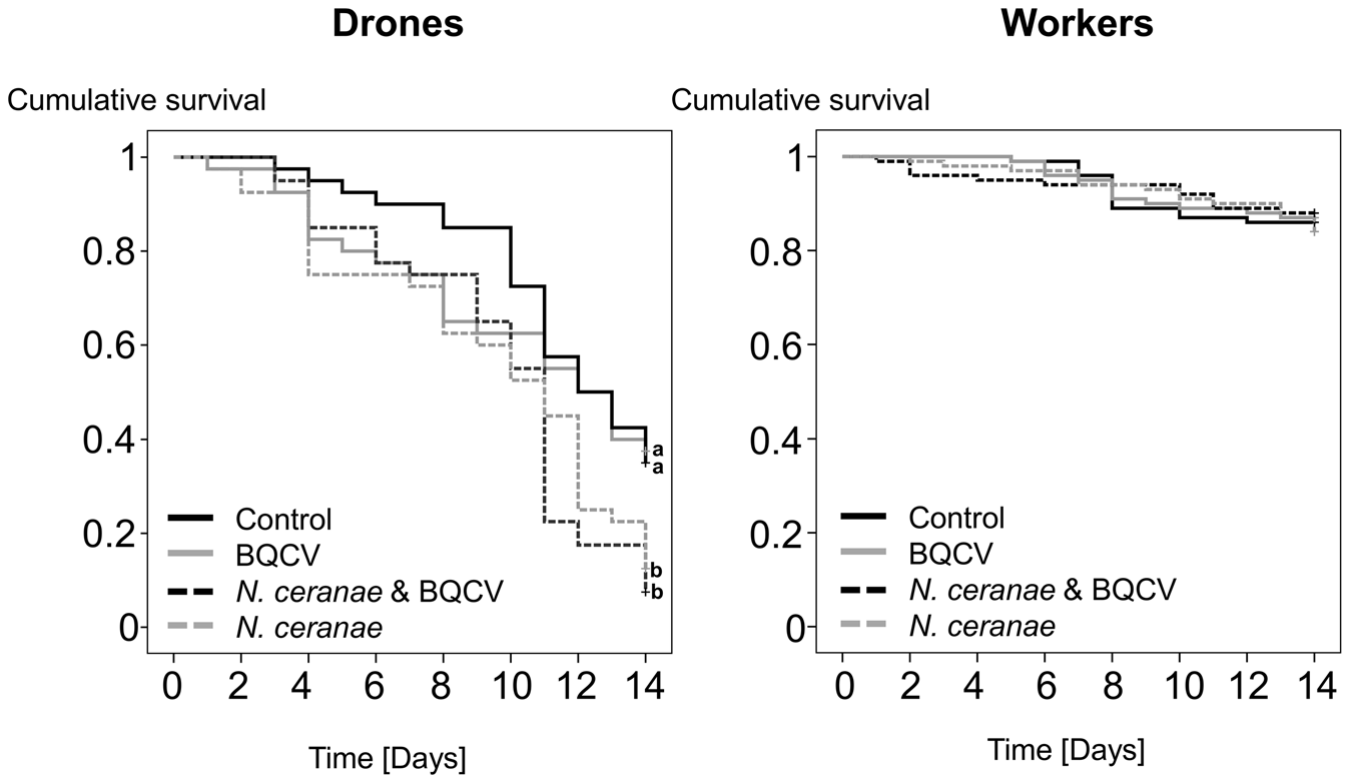
Cumulative survival of drones and workers during the 14 days of the cage trial. Bees that were terminated on day 14 were treated as censured in the analyses. Drones showed significant higher mortality in the treatment groups that were challenged with *N. ceranae* (*P*s = 0.037), indicated by different letters (a, b) in the figure. Workers of the different treatment groups showed no differences in mortality. The groups with BQCV-inoculation differ from the other groups in terms of treatment from day 7 onwards.

Drones inoculated with *N. ceranae* spores, regardless of co-inoculation with BQCV, had significantly greater mortality than those not inoculated with *Nosema* (Kaplan-Meier Log-Rank, both *P*s<0.04), whereas worker mortality did not differ significantly among treatment groups (Kaplan-Meier Log-Rank, *P* = 0.89; Fig. 1). In all treatment groups, 12 to 16% of the workers died during the experiment, whereas drone mortality rates were much higher at 62.5% (control), 65% (BQCV), 87.5% (*N. ceranae*) and 92.5% (*N. ceranae* & BQCV). Overall, drone survivorship was significantly lower than workers (Kaplan-Meier Log-Rank, all *P*s<0.001; Fig. 1).

**Figure 2:**
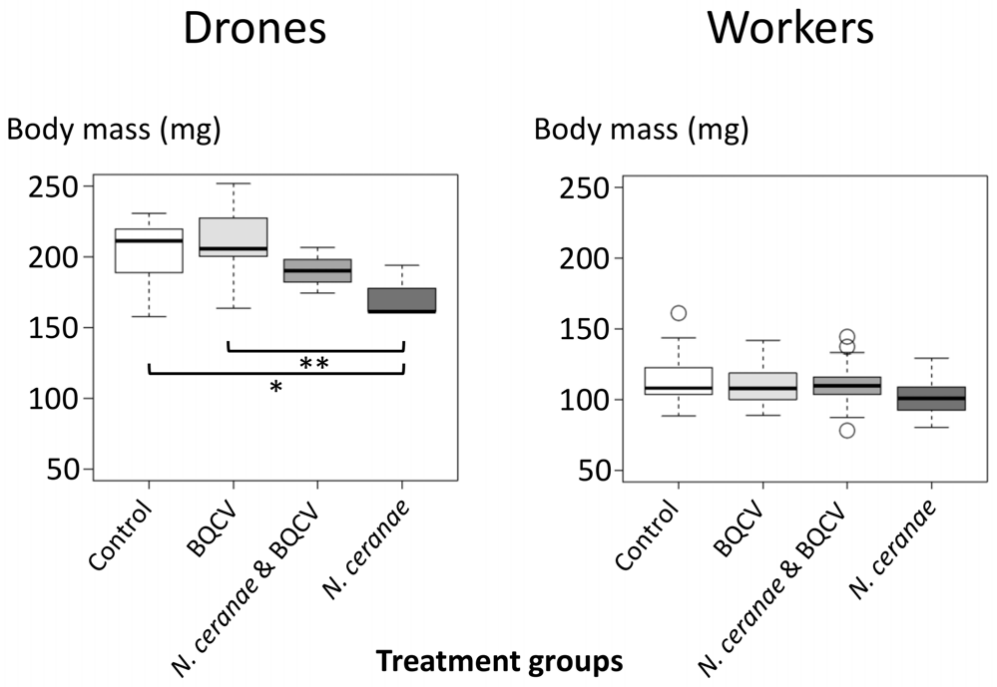
Body mass of drones and workers of the four treatment groups on the last day of the cage trial after 14 days. The boxplots show interquartile range (box), median (black line within the interquartile range), data range (dashed vertical lines) and outliers (open dots). Significant differences (all Ps<0.05) were detected between the *N. ceranae* and control (*  = P<0.05) as well as *N. ceranae* and BQCV (**  = P<0.01) group in the drones as indicated by black horizontal bars between the respective treatment groups under the boxplots. No differences were detected between the treatment groups of the workers.

The mean body mass of *N. ceranae* inoculated drones was 170.78 mg compared to 190.27 mg for *N. ceranae* & BQCV co-inoculated drones, while the control and BQCV-inoculated drones were 202.43 and 211.4 mg on average, respectively (Table 2). The mass of drones inoculated with *N. ceranae* spores alone was significantly lower than the control and BQCV treatment groups (Tukey's HSD, both *P*s<0.05), but not significantly less than the mixed *N. ceranae* & BQCV treatment group (Tukey's HSD, *P* = 0.63; Fig. 2). No significant difference in drone mass was observed among control, BQCV, or *N. ceranae* & BQCV treatment groups (Tukey's HSD, all *P*s>0.45). For the worker experiment, mean body mass ranged from 103.06 mg (*N. ceranae*) to 112.97 mg (control) for all treatment groups and did not significantly differ among the groups (Tukey's HSD, all *P*s>0.45; Fig. 2 and Table 2).

**Table 2:** Details of measured body mass of worker and drones that survived until the end of the experiment on day 14.

Treatment group	Mean body mass [mg]	Standard error (SE)	Median body mass [mg]	Range [mg]
**Drones**				
Control	202.43	5.91	211.2	72.9
BQCV	211.4	6.67	205.7	88.1
*N. ceranae* & BQCV	190.27	9.24	190	32
*N. ceranae*	170.78	6.62	161.2	33.7
Workers				
**Control**	112.97	3.57	108.3	72.5
BQCV	110.64	2.43	108.2	52.8
*N. ceranae* & BQCV	110.21	3.12	110	66
*N. ceranae*	103.06	2.75	101	49

### Nosema ceranae quantification

**Figure 3:**
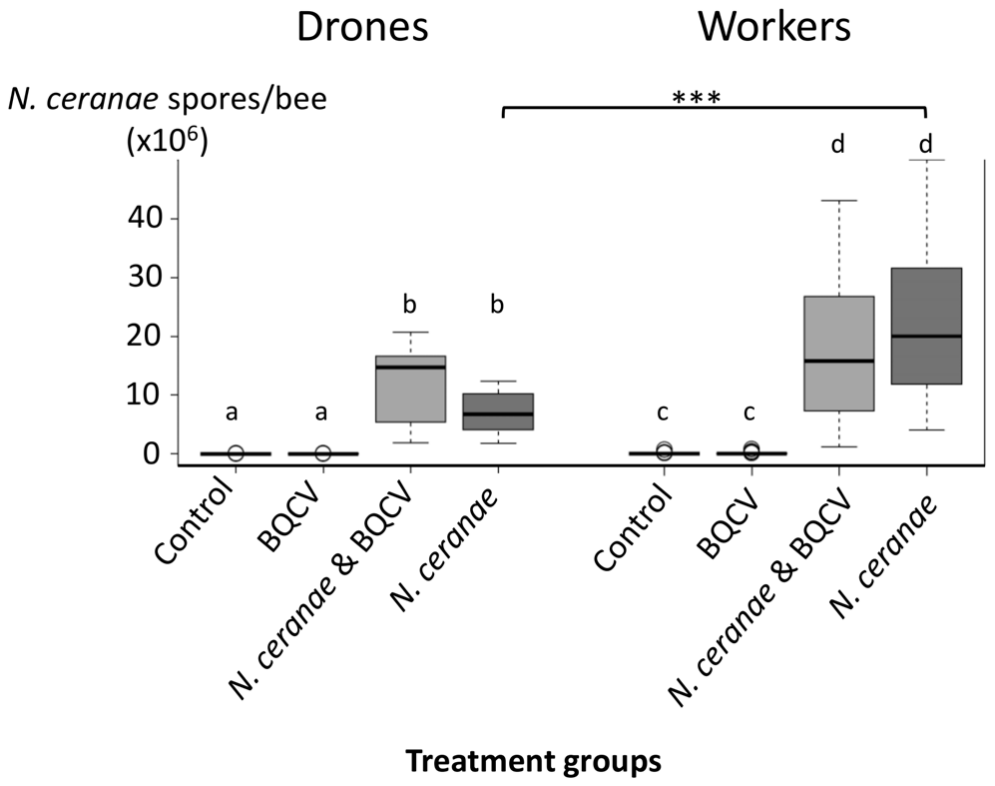
*N. ceranae* spores per bee in drones and workers of the four treatment groups after termination of the bees on day 14. The boxplots show interquartile range (box), median (black line within the interquartile range), data range (dashed vertical lines) and outliers (open dots). Significant differences within the bee type (drones and workers) are marked through different letters. Significant differences between the treatment groups of workers and drones are indicated by a black horizontal bar (***  = *P*<0.001).

All individuals (workers and drones) that were experimentally inoculated with *N. ceranae* spores developed detectable *Nosema* infections. At 14 d after inoculation with *N. ceranae*, drones had mean spore amounts of 6.93×10^6^ (SD (standard deviation): 3.88×10^6^) spores per bee in the *N. ceranae* group and 11.62×10^6^ (SD: 7.28×10^6^) spores per bee when inoculated with both pathogens. Spore amounts in *N. ceranae* inoculated workers were 19.43×10^6^ (SD: 11.47×10^6^) spores per bee in the *N. ceranae* group and 16.6×10^6^ (SD: 11.11.×10^6^) in the *N. ceranae* & BQCV group, respectively. A small percentage of the non-inoculated drones (<16%) and workers (<24%) had low levels of spore amounts. The mean spore values of the non-inoculated drones were 0.0156×10^6^ (SD: 0.044×10^6^) spores per bee for the controls and 0.0154×10^6^ (SD: 0.043×10^6^) for the drones that were inoculated with BQCV only. For the workers, the controls showed mean spore amounts of 0.05×10^6^ (SD: 0.13×10^6^) spores per bee and the BQCV-inoculated workers 0.064×10^6^ (SD: 0.16×10^6^) spores per bee (Fig. 3). No differences in spore amounts were observed among treatment groups that were inoculated with *N. ceranae*, regardless of BQCV inoculation for both drones and workers (Tukey's HSD, all *P*s>0.1). Drones inoculated with *N. ceranae* but not with BQCV showed significantly lower pathogen intensities than workers from the same treatment group (ANOVA, *P*<0.01; Fig. 3). The comparison of spore amounts in drones and workers revealed higher spore loads in workers in the *N. ceranae* treatment (Tukey's HSD, P<0.001, Fig. 3), but no differences in the other treatments.

### BQCV quantification

**Figure 4:**
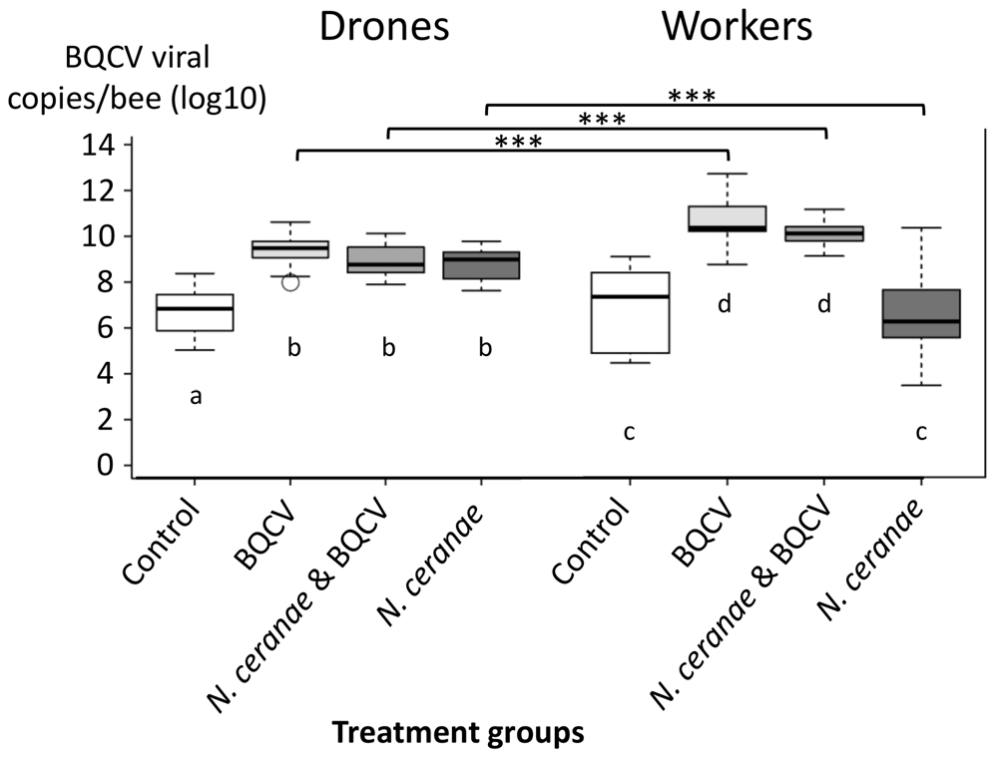
BQCV loads expressed as viral copies per bee (log10-transformed) of drones and workers of the four treatment groups after termination of the bees on day 14. The boxplots show interquartile range (box), median (black line within the interquartile range), data range (dashed vertical lines) and outliers (open dots). Significant differences within the bee type (drones and workers) are marked through different letters. Significant differences between the treatment groups of workers and drones are indicated by black horizontal bars (***Ps<0.001).

Drones inoculated with BQCV only showed an average (±SD) of 10^9.23±0.64^ viral copies per bee, while those infected with both, *N. ceranae* and BQCV showed an average of 10^8.81±0.77^ viral copies per bee. Non-BQCV-inoculated drones of the *N. ceranae* only group exhibited an average of 10^8.42±1.2^ viral copies and the control drones 10^6.71±0.96^ viral copies per bee, respectively. The mean viral copies of BQCV per bee in the workers were in the range of 10^9.91±0.48^ for workers that were inoculated with *N. ceranae* and BQCV, to 10^10.44±0.86^ copies per bee for workers that were inoculated with BQCV only. The mean viral copies per bee for the workers that were not inoculated with BQCV was 10^6.49±1.77^ viral copies in the *N. ceranae* only group to 10^6.55±1.68^ viral copies per bee in the control group. At 14 d drones from the control treatment exhibited significantly lower BQCV loads than other treatment groups (Tukey's HSD test, all *P*s<0.001); no difference was observed among BQCV alone, *N. ceranae* & BQCV, and *N. ceranae* alone treatments (Tukey's HSD test, all *Ps*>0.55; Fig. 4). For the worker experiment at the same time period, viral load was significantly greater in treatments inoculated with BQCV versus those that were not (Tukey's HSD test, all *Ps*<0.001), however, there was no difference in viral loads between treatments inoculated (BQCV versus *N. ceranae* & BQCV, Tukey's HSD test, *P*>0.49) or not inoculated with virus (Control versus *N. ceranae*, Tukey's HSD test, *P*>0.99). Significantly higher viral loads were observed in workers inoculated with BQCV compared to drones (ANOVA, all *P*s<0.001; Fig. 4) apart from for the *N. ceranae* treatment group (ANOVA, *P*<0.001).

### Inter-specific interactions

No correlation was observed between the amounts of BQCV and *N. ceranae* in either drones or workers inoculated with both pathogens (Pearson Correlation, workers: *R* = −0.048, *P* = 0.82; drones *R* = 0.177, *P* = 0.704).

## Discussion

The data imply that there are sex-specific differences in honey bee susceptibility to the pathogens *Nosema ceranae* and, to a lesser degree, BQCV, as indexed by mortality, body mass and infection level. During the 14-day study period, drones suffered elevated mortality and those surviving had reduced body mass due to *N. ceranae* infection, but not BQCV infection, while worker mortality and body mass was unaffected by either *N. ceranae* or BQCV. Surviving drones also contained lower titres of both *N. ceranae* and BQCV than surviving workers, while there was no evidence of any specific interaction between *N. ceranae* and BQCV in these experiments: neither *N. ceranae* nor BQCV was particularly affected by co-infection with the other pathogen, in both drones and workers. These global findings support previous studies demonstrating differences between honey bee drone and worker susceptibility to pathogens [45]–[46], [59]–[60], [71], as well as similar studies on bumble bees (*Bombus* spp.) that observed no clear trend of sex-specific pathogen susceptibility differences [10]. Previous reports of a co-dependent interaction between *N. apis* and BQCV [46] were not replicated here with *N. ceranae* and BQCV. However, these observations have to be interpreted within the context of the greater overall sensitivity of drones to the laboratory hoarding cage conditions, compared to the workers, as reflected by their different control mortalities during the 14 day trial period. This increased sensitivity and reduced lifespan has been observed repeatedly for drones maintained under artificial conditions (e.g. [54], [72]). In fact, the drones in this study showed considerably better control survival than previously reported (e.g. [54], [72]). Workers also survive less well in hoarding cages than in a natural colony environment, but are much more resilient to these conditions than drones. This reflects that drones and workers react differently to changes in their environments, and that hoarding cages are a more stressful environment (for both) than the natural colony environment. However, these (unavoidable) limitations to the experimental system used here have significant bearing on how the data may be interpreted, and pose clear corresponding limitations on how far they can be extrapolated. Broadly regarded, the observation that within the 14 day period of the experiment drones experience greater levels of stress in hoarding cages than workers, as evidenced by their greater background mortality, may also make them more susceptible to pathogens such as *N. ceranae* and BQCV. If the workers were investigated under conditions generating a similar background control mortality as for the drones here (for example, by extending the window of observation), they too might show elevated mortality due to pathogen infection. However, the existential differences between drones and workers and their relationship with stress will also affect their relationships with the pathogens that they share, which may ultimately be attributable to their very different roles for colony functionality [73] and/or ploidy levels [9]. Below we discuss each of the major findings of this study in turn, and interpret them within the contexts outlined above, and the limitations set by the study conditions.

*Nosema ceranae* showed a strong effect on drones in the present study. Even though the overall mortality was higher in the drones, the impact of *N. ceranae* on drone survival in cages was significant, thereby highlighting the strong effect this midgut parasite can have on male bees. Consistent with the mortality data, *N. ceranae* infection also resulted in lower body mass in drones, which constitutes another commonly measured indicator for the health status of honey bees (e.g. [17]) and further confirms the considerable impact of *N. ceranae* on drones.

*Nosema ceranae* parasitism in drones has not yet been closely investigated. Natural infections occur in both immature and mature drones [74], and population-specific differences in host survivorship as a response to parasitism have been observed [53]. Our experiments confirmed that *N. ceranae* can successfully invade and reproduce in drones, and that parasitism can result in increased host mortality. Interestingly, fewer than 2 million spores 8 days post-infection results in reduced survivorship [53]. Our results also suggest that drone mortality due to *N. ceranae* infection can occur within one week of initial parasitism.

Numerous studies have investigated effects of *N. ceranae* in honey bee workers under *in vitro* conditions. In this study, no effect of *N. ceranae* parasitism on worker mortality or body condition was observed, whereas other studies have demonstrated varying levels of pathogenicity (e.g. [75]–[79]). These differences could be the result of various genetic [49], environment [33], or methodological [58], [61] influences. The same factors may be responsible for differences in infection levels observed among studies. We observed spore amounts in the workers (∼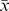 = 16–20 million spores per individual 14 d post-infection) that were similar to previous studies at approximately the same period post-infection (e.g. [33], [75]), but not all (e.g. [34]).

The comparison of observed spore amounts in drones and workers of this study revealed significantly higher levels in workers than drones, demonstrating a sex-specific difference in infection level susceptibility. Interestingly, no sex-specific difference in susceptibility was observed for *N. apis* when measured as parasite prevalence in field-collected bees [46]. This highlights the importance of type of study (laboratory or field), type of infection or susceptibility measurement. For example, Higes et al. (2008) argue that infection prevalence constitutes a better indicator for colony disease from *N. ceranae* infection than mean infection level [80]. Differences in *N. ceranae* susceptibility between the sexes could be due to allelic variation associated with haplo-diploidy [9], or because of distinct resource investment strategies that influence resistance to disease [8]. Another contributing factor may be differential immune responses to disease by drones and workers. Drones from honey bee lineages selected for *Nosema* tolerance have an up-regulated immune response, suggesting that the *Toll* pathway is important for defence against *N. ceranae* [53]. However, *N. ceranae*-infected workers may suffer from immunosuppression through down-regulation of genes that are part of the honey bee's humoral (defensin, abaecin, apidaecin and hymenoptaecin) and cellular (glucose dehydrogenase) immune system [50]–[51]. The sex-specific differences in the measured infection levels may also be explained by general physiological differences between drones and workers [37] that could influence the dynamics of infection development and thereby the infection level at any given point in time. Indeed, it may be the case that the infection develops differentially in drones and workers over time for a variety of reasons, and that this might be a potential explanation for the differences in the titres since we looked at both drones and workers at the very same day. A further explanation for lower titres in surviving drones may actually be that higher titres would have killed them. Since infected workers do not show an increased mortality, they can tolerate higher pathogen titres, thereby also confirming differences in pathogen susceptibility.

Contrary to *N. ceranae*, inoculation with BQCV had no impact on the mortality or body mass of drones or workers. BQCV is a very common virus with a broad geographic distribution in European honey bees (e.g. [52], [81]–[82]), therefore it was not surprising that low levels of virus were detected in our non-inoculated bees. Very little is known of how BQCV affects individual drone and worker honey bees. It may damage and even kill developing drone [83] as well as queen larvae [84] during natural infections, but no information is currently available about potential effects of BQCV on adult bees, either drones or workers. In workers, detectable BQCV infections do not cause visible symptoms [52]. At the colony level, infection could not be linked to Colony Collapse Disorder in the United States [20], although it was associated with reduced populations in Israel [85]. Our data suggest that oral inoculation of BQCV, at the titres employed here, does not affect mortality and body mass of drones or workers. However, it is possible that such effects could occur at higher inoculum titres, or when acquired through a different transmission route. Brødsgaard et al. (2000) demonstrated that pathogenicity of ABPV was influenced by route of transmission [86]. Unfortunately, few studies report quantities of BQCV or threshold levels for symptoms. Those that are available employed different sample types (e.g. pooled bees from a colony in Gauthier et al. 2007 [87]) or experimental set-up (e.g. natural infection as in Locke et al. 2012 [65]) that makes direct comparisons difficult and constitutes a potential explanation for the considerable differences in reported levels. While Gauthier et al. (2007) reported a mean of BQCV and ABPV of 1.52×10^8^ equivalent copies per adult bee, Locke et al. (2012) displayed values in the range of 10^4^ to 10^8^ copies per bee. In the present study, median BQCV copies of inoculated bees were in the range of 10^7^ to 10^10^ and therefore in line with reported field levels under natural conditions [65], [87]. Similar to *N. ceranae*, pathogen levels of laboratory studies using artificial infections tend to be higher than field levels of pooled samples, due to the absence of potential dilution effects (e.g. [34] vs. [88]).

Workers from treatments inoculated with BQCV showed higher numbers of viral copies per bee than drones. Similar to *N. ceranae*, this result reveals sex-specific differences in infection level susceptibility of honey bees to BQCV. The higher infection levels of both pathogen and parasite in the workers contradict the initial hypothesis of drones being parasitized more intensively than workers. Potential reasons for the higher pathogen levels in the workers may be similar to those already mentioned for *N. ceranae* above. In contrast to Bailey et al. (1983) [25], our data suggest successful BQCV infection following oral inoculation regardless of *N. apis* or *N. ceranae* infection.

Our results do not suggest a close relationship between *N. ceranae* and BQCV. Unlike *N. apis* and BQCV [25], presence of *N. ceranae* did not promote infection of the virus in either drones or workers, despite inoculation by the former occurring 7 days prior that resulted in increased host mortality likely from gut tissue damage [49]. Elevated BQCV quantities in the *N. ceranae* only treatment group resulted from two outliers. Because natural covert bee virus infections are common [89], even in the control groups, it is likely that these highly infected individuals were included by chance as no other data support this positive association. Furthermore, no correlation was observed between quantities of *N. ceranae* and BQCV in either drone or worker groups that were co-inoculated with both pathogens. Lack of a positive numeric response to co-infection could be explained by differences in host immune responses to *N. ceranae* compared to *N. apis* that in turn may influence susceptibility to BQCV. Conversely, a lack of negative response could result from *N. ceranae* and BQCV infections below respective carry capacities (e.g. for *N. ceranae* [34]), suggesting that competition for limited resources did not occur. Although co-infection resulted in significantly greater drone mortality compared to individuals inoculated by BQCV alone, no difference was observed between individuals co-infected and those parasitized by just *N. ceranae*. This suggests that the microsporidian parasite, and not co-infection or BQCV, influenced host survival, and is similar to results reported by Otteni and Ritter (2004) that found *N. apis* affected worker survival compared to ABPV [90].

Our data clearly demonstrated that under the experimental conditions honey bee drones are more susceptible to *N. ceranae* when the indices of host mortality and body condition are used. This highlights the importance of carefully choosing measures of susceptibility during evaluations, as well as the need to further study the influence of parasites and pathogens on drones due to their contribution to queen fertility. Future investigations should not only focus on understanding the influence of stress on drones, and how this may result in overall decreased colony health and fitness, but also on further improving methods for maintaining drones in the laboratory.

The observation that *N. ceranae* inoculation leads to higher mortality and lower body mass only in drones, despite lower spore amounts relative to those found in workers, clearly demonstrates increased susceptibility of the males. This particular outcome is in line with the haploid susceptibility hypothesis, stating higher susceptibility in haploid males due to hemizygosity at immune loci [9]. Nevertheless, further work is needed to determine the mechanisms responsible for *N. ceranae* defence by both drones and workers to truly understand why differences may occur. Because of the importance of immunity, particularly to *N. ceranae* infection [50]–[53], [91], comparative studies investigating host immune response are prudent.
